# Motor Neglect and Future Directions for Research

**DOI:** 10.3389/fnhum.2013.00110

**Published:** 2013-03-28

**Authors:** Dimitrios S. Sampanis, Jane Riddoch

**Affiliations:** ^1^School of Psychology, University of BirminghamBirmingham, West Midlands, UK; ^2^Experimental Psychology, University of OxfordOxford, UK

Here we present an opinion on “motor neglect,” one of the several scotomas in neglect research (Kerkhoff and Schenk, 2012). We describe what it is, outline its anatomical substrate, and its frequency in a stroke population. We outline evidence to suggest that motor neglect reflects the impaired ability to generate movements and discuss a possible rehabilitation technique which may target this particular deficiency. We feel that it is a timely “opinion.” Motor neglect may occur in the absence of visuospatial neglect (Laplane and Degos, 1983; Punt et al., 2005) and it can have a severe and detrimental effect on rehabilitation outcomes (Siekierka-Kleiser et al., 2006).

“Motor neglect,” a term originally coined by Laplane and Degos (1983), refers to the underutilization of the affected limb compared to the healthy one following brain damage despite normal muscle strength, reflexes, and sensation. It may be distinguished from “directional hypokinesia” (originally described by Heilman et al., 1985) referring to slowness in the initiation of contralesional movements, reduced spatial exploration toward the contralesional side, and insufficient amplitude of contralesional limb movements. Patients with motor neglect typically underuse the contralesional side (even where this involves inconvenience); have little or no involvement of the contralesional limb in bimanual tasks (e.g., clapping, opening a bottle); have little or no involvement of the contralesional limb when automatically gesturing; however, they have relatively normal movement when encouraged *specifically* to use the contralesional limb (Laplane and Degos, 1983; Punt and Riddoch, 2006; Garbarini et al., 2012a,b). Unlike patients with hemiplegia, patients with motor neglect have no paresis, no increase in muscle tone, no pyramidal signs, or alterations in sensation (von Giesen et al., 1994). There is relatively no information on how these patients are able to manage their activities of everyday living. Laplane and Degos (1983) suggest that increased determination on the part of the patient results in tasks eventually being performed (they describe patients with right hemisphere lesions using verbal strategies, while with left hemisphere lesions patients become “left-handed”).

There are differential reports as to the frequency of motor neglect. Siekierka-Kleiser et al. (2006), report an incidence of 33% incidence in an acute stroke population with 74% of the motor neglect sample having right hemisphere lesions, while Buxbaum et al. (2004) report an incidence of 12% in an acute and 8% in a chronic stroke population (all patients in the Buxbaum study had right hemisphere lesions). According to Siekierka-Kleiser et al., patients with motor neglect show poor motor recovery over the first 7 days post-stroke relative to the patients without motor neglect; although a sub-group (26.3%) recovered well, and two of the sub-group had left hemisphere lesions.

von Giesen et al. (1994), using positron emission tomography (PET) with four patients with motor neglect, demonstrated that while primary areas underlying the motor output system (the primary sensorimotor cortex, basal ganglia, and cerebellum) were unimpaired, there was poor glucose uptake in premotor, prefrontal, parietal, and cingulate cortex areas, as well as the thalamus. This substantiates the clinical manifestation of normal muscle strength, reflexes, and sensation in motor neglect. von Giesen et al. hypothesized that the intact motor cortical output system is deprived of sensory information and the voluntary drive needed for movement execution (see also Laplane and Degos, 1983).

Recent evidence implicating the parietal regions for movement generation comes from Desmurget et al. (2009). They contrasted the effects of direct stimulation of parietal and premotor regions. Stimulation of inferior parietal regions (IPL) produced a desire to move without any overt movement being produced or EMG activity recorded in the concerned muscles. If the intensity of stimulation was increased, patients reported that movement had occurred; however, again, no actual movement or EMG activity was observed. Desmurget et al. argue that the “wanting to act feeling,” resulting from IPL stimulation, is indicative of intentions to move generated before any motor act (Desmurget and Sirigu, 2009, 2012; Desmurget et al., 2009). Sirigu et al. (2004) have also shown that lesions to the parietal lobe (involving the angular gyrus in particular) result in deficits in the subjective experience of wanting to move in a task where patients were free to execute a movement at a time of their own choosing. Thus, behaviorally, control participants demonstrated an anticipatory period prior to the actual movement, parietal patients reported the desire to movement at a time which was very close to the actual time movement was initiated.

The inability to generate actions in motor neglect is illustrated in a recent study. Garbarini et al. (2012a,b) contrasted the performance of patients with motor neglect (and a lack of voluntary drive to initiate action but intact ability to execute motor acts) with patients with anosognosia (who show the reverse deficit, intact voluntary drive but impaired motor ability). While blindfolded, the patients had to draw circles and lines, either performing unimanual drawing movements (the right hand drew unilateral lines) or bimanual movements (the right hand drew lines and simultaneously, the left hand drew circles). They showed that bimanual spatial coupling, as found in normal subjects is not present in patients with motor neglect, although such coupling was preserved in the anosognosic hemiplegic patients. This is a particularly striking finding given that anosognosic patients are unable to move the contralesional limb, while that ability is intact in patients with motor neglect.

As yet (as far as we know) there have been no studies specifically addressing rehabilitation for motor neglect. Exciting new techniques such as repetitive TMS and tDCS (used either to enhance the activity in the lesioned hemisphere or at suppressing the over-activity observed in the unaffected hemisphere) have been used for visuospatial neglect *in general* but not for motor neglect *in particular*. Thus, while suppressing over-activity in the contralesional hemisphere may facilitate ipsilesional performance, it is not clear how it may benefit contralesional action planning. Increasing the activity in lesioned hemisphere may not improve performance – Desmurget et al. (2009) report no benefit of increasing stimulation of IPL on movement generation. Rehme and Grefkes (2013) have argued that the best predictor for good recovery from stroke *in general* (from the acute phase to the chronic phase) is an increase of the coupling between ipsilesional premotor areas (supplementary motor area, ventral premotor cortex, and ipsilesional M1). Such coupling may be critical in patients with motor neglect. Recent studies suggest that noradrenergic (NA) stimulation may be the tool for the job. Grefkes et al. (2010) used a crossover design where healthy subjects were stimulated using the selective noradrenaline reuptake inhibitor reboxetine (RBX) or a placebo. The participants performed goal directed movements with a joy-stick. Drug-related changes in blood oxygen level – dependent activity and interregional connectivity were assessed using functional magnetic resonance imaging (fMRI) and dynamic causal modeling (DCM). The results showed that movement speed increased as a result of RBX (with a corresponding increase in regional activation), and that there were also complex network effects affecting both neural processing within and across the hemispheres. Within the right hemisphere, there this was enhanced activity in areas known to be involved in visuospatial attention and motor control (see Corbetta and Shulman, 2002). In addition, there was increased coupling of the right V1, IPS, and FEF/dPMC with left hemispheric areas, which was independent from task difficulty. Grefkes et al. suggest that the activation reflects enhanced engagement of transformation processes facilitating the integration of visual information into planned motor programs. Subsequently, Wang et al. (2011) studied the effects of NA stimulation at behavioral and neural levels using fMRI in sub acute patients. DCM was applied to fMRI data from key motor areas to assess the effects of NA stimulation on interregional connectivity within the cortical motor system. The results showed a reduction of cortical “hyperactivity” toward physiological levels observed in healthy control subjects, especially in the ipsilesional ventral PMC and SMA, but also in the TPJ and prefrontal cortex. Together these studies suggest that NA stimulation may help to modulate the pathologically altered motor network architecture in stroke patients, resulting in increased coupling of ipsilesional motor areas and improving motor function. Future studies may show NA stimulation to be of significance in patients with motor neglect showing impaired attention and visuomotor intention particularly in the acute phase of stroke when disconnectivity between motor areas is greatest (Rehme et al., 2011). As the time course of spontaneous neurological recovery of neglect as well as motor impairment shows a natural logistic curve up to the first 12–14 weeks post-stroke, after which severity becomes invariant (Kwakkel et al., 2004; Nijboer et al., 2012), NA stimulation may be most beneficial within this time-window in the facilitation of natural recovery.
